# Neural Plasticity Associated with Hippocampal PKA-CREB and NMDA Signaling Is Involved in the Antidepressant Effect of Repeated Low Dose of Yueju Pill on Chronic Mouse Model of Learned Helplessness

**DOI:** 10.1155/2017/9160515

**Published:** 2017-09-17

**Authors:** Zhilu Zou, Yin Chen, Qinqin Shen, Xiaoyan Guo, Yuxuan Zhang, Gang Chen

**Affiliations:** Center for Translational Systems Biology and Neuroscience and Key Laboratory of Integrative Biomedicine for Brain Diseases, Nanjing University of Chinese Medicine, Nanjing 210023, China

## Abstract

Yueju pill is a traditional Chinese medicine formulated to treat syndromes of mood disorders. Here, we investigated the therapeutic effect of repeated low dose of Yueju in the animal model mimicking clinical long-term depression condition and the role of neural plasticity associated with PKA- (protein kinase A-) CREB (cAMP response element binding protein) and NMDA (N-methyl-D-aspartate) signaling. We showed that a single low dose of Yueju demonstrated antidepressant effects in tests of tail suspension, forced swim, and novelty-suppressed feeding. A chronic learned helplessness (LH) protocol resulted in a long-term depressive-like condition. Repeated administration of Yueju following chronic LH remarkably alleviated all of depressive-like symptoms measured, whereas conventional antidepressant fluoxetine only showed a minor improvement. In the hippocampus, Yueju and fluoxetine both normalized brain-derived neurotrophic factor (BDNF) and PKA level. Only Yueju, not fluoxetine, rescued the deficits in CREB signaling. The chronic LH upregulated the expression of NMDA receptor subunits NR1, NR2A, and NR2B, which were all attenuated by Yueju. Furthermore, intracerebraventricular administration of NMDA blunted the antidepressant effect of Yueju. These findings supported the antidepressant efficacy of repeated routine low dose of Yueju in a long-term depression model and the critical role of CREB and NMDA signaling.

## 1. Introduction

Major depressive disorder (MDD) is a state of low mood and aversion to activity that can affect a person's thoughts, behavior, feelings, and sense of well-being [1]. MDD afflicts approximately 16 percent of the world's population at some point in their lives [2, 3]. Although a number of antidepressants, such as the first-line selective serotonin reuptake inhibitors (SSRIs), are available, a remarkable population of patients never attain sustained remission of their symptoms [4, 5]. These and other disadvantages such as delayed onset of efficacy of SSRIs challenge the traditional monoamine-based hypothesis of depression, and emerging evidence favors the neural plasticity hypothesis which proposes an important role of the impaired neural plasticity including neurotrophic factors, cAMP response element binding protein (CREB) signaling, synaptic plasticity influenced by N-methyl-D-aspartate (NMDA) signaling, adult neurogenesis in depression, and neural plasticity as the crucial targets for antidepressant action [6, 7].

CREB signaling, activated by one of the classic upstream activator protein kinase A (PKA), regulates expression of genes that promote synaptic and neural plasticity, including proteins for spine formation [8, 9]. Both human and experimental studies supported the link of PKA-CREB signaling to depression and its treatment [10]. Brain-derived neurotrophic factor (BDNF) is one of the best studied neurotrophic factors implicated in depression and antidepressant effect [11]. Activation of PKA-CREB signaling is also capable to upregulate BDNF expression. Additionally, Increasing number of studies suggest *N*-methyl-D-aspartate (NMDA) receptors (NMDAR) are prefoundly associated with depression [12, 13]. NMDARs are glutamate ionotropic receptors that play an important role in synaptic transmission and plasticity. Some NMDAR antagonists were identified to rapidly induce antidepressant effect by instant upregulation of expression of BDNF and spine formation [14, 15]. Therefore, enhancement of neural plasticity can result from activation of PKA-CREB or inhibition of NMDA signaling.

The learned helplessness (LH) procedure is one of the validated animal models of depression, extensively used to model stress-induced depression-like behavior in rodents [16–19]. It represents a model with good face, construct, and predictive validity [20] and has been shown in subpopulation of MDD patients. In the model, animals are exposed to unpredictable and uncontrollable stress, such as electroshocks, and then develop coping deficits in aversive but escapable situations [21]. As a relatively short and reliable stress-induced model, a variety of LH procedures have been developed. However, few of previous studies investigated the validity of a model with long-term depressive-like symptoms, which is a typical and obligatory feature for clinical depression. Long-term model is relatively more informative to uncover the mechanisms of the disorder and long-term efficacy of antidepressants.

To develop novel antidepressant therapy, some attention has been paid to integrative medicine [22]. Yueju pill, a traditional Chinese herbal medicine formulated 800 years ago to treat mood disorders related syndromes has been used to treat MDD and contain multiple compounds with antidepressant potential [23–25]. More recently, a relative high dose of Yueju demonstrated rapid antidepressant efficacy in both preclinical and clinical studies [26, 27]. However, as a nonprescription drug in oriental medicine, the routine dose of Yueju has not been tested scientifically for antidepressant effect previously. Here, we developed a long-term learned helplessness depression model to test the therapeutic effects of Yueju. Additionally, PKA-CREB and NMDA signaling, as well as BDNF was examined in the mice subjected to routine dose of Yueju following chronic learned helplessness procedure. Finally, the role of NMDA signaling in Yueju's action was further verified by using intracerebroventricular pharmacological infusion approach.

## 2. Materials and Methods

### 2.1. Animals

Male and female Kunming mice were obtained from the China Academy of Military Medical Sciences (Beijing). Mice aged approximately 6 weeks old (18–24 g) were habituated to animal facilities for 7 days prior to behavioral testing. All animal experiments were in accordance with the Guide for the Care and Use of Laboratory Animals approved by the Institutional Animal Care and Use Committee at Nanjing University of Chinese medicine.

### 2.2. Drugs

Fluoxetine (Sigma-Aldrich, St. Louis, MO, USA) was dissolved in 0.9% saline. The OCT Yueju pills (Jiangsu 707 Natural Pharmaceutical Co. Ltd., lot number 150801) were ground into powder and dissolved with 0.9% saline with different doses. The solutions of the Yueju, fluoxetine, and vehicle were administered to the mice via intragastric administration, and the concentration of the solution was 1 mg/ml. The dose for fluoxetine was 18 mg/kg/day, and N-Methyl-D-aspartic acid (Sigma-Aldrich, St. Louis, MO, USA) was administered by intracerebroventricular infusion (i.c.v.), 1 pmol/3 *μ*l. All drugs were dissolved in 0.9% saline.

### 2.3. Behavioral Tests

All behavioral tests were performed during the late light phase. Animals were transferred to the testing room and habituated to the room conditions for at least 1 hour before the beginning of the behavioral experiments. Behavioral testers were blinded for experimental groups.

#### 2.3.1. Open Field Test

Mice were placed in individual open field arena (40 × 40 × 40 cm) and allowed to freely explore for 2 hours. A camera was mounted above the open box for recording locomotor activity. The entire test arena was adjusted to even illumination. Mice were placed in the center of the arena and all owed to freely explore for 5 minutes. The total distance traveled and time spent in the central area was measured. The testing apparatus was thoroughly cleaned before each animal using 70% ethanol.

#### 2.3.2. Tail Suspension Test

The apparatus is consisted of four chambers. The front of the box was open, and a bar was placed horizontally 1 cm from the top with an attached vertical bar hanging down in the center. Mice were individually suspended 1 cm from the tip of the tail to the vertical bar with adhesive tape. A camera positioned in front of the TST box was used to record the animals' behavior for 6 minutes. The software analysed the Immobility time of the last 4 minutes. Mice were returned in individual cages and remained so until the end of the experiment.

#### 2.3.3. Forced Swim Test

Mice were placed in a clear Plexiglas cylinder (25 cm high; 10 cm in diameter) filled to a depth of 10 cm with 23–25°C water. A camera recorded the 6-minute swim session. The immobility of the mice was measured during the last 4 minutes of the test. Immobility in this test was defined as they floated passively in water.

#### 2.3.4. Novelty-Suppressed Feeding Test

After fasting for 24 hours, the mice were habituated for 2 hours in the experimental environment and placed in a new cage. The center of the cage placed an already weighed grain. Each mouse was placed in a corner of the box and allowed to explore for up to 10 minutes. The trial ended when the mouse chewed a part of the chow. The amount of food consumed in the home cage was taken as the weight of chow consumed in 10 minutes. Food consumption is the weight of chow consumed divided by the weight of the mice. The time the mouse first started to consume the food pellet was recorded as the latency.

#### 2.3.5. Learned Helplessness (LH) Test

LH consists of two stages: training stage and testing stages.

In the training stage, regular LH paradigm was carried out as described previously [28]. LH was performed in a shuttle cage (40 × 10 × 13 cm) that was divided equally into two chambers. Learned helplessness was induced in mice by administering 120 scrambled, inescapable foot shocks (0.45 mA shock amplitude, 15 s duration, 18–44 s average interval) over a 1 h session. For chronic LH paradigm, mice were trained for 3 consecutive days as described, followed by two additional intermittent training on day 8 and day 13. Control animals were exposed to the apparatus for the same period without receiving foot shocks.

In the testing stage, each mouse was given 30 shuttle escape trials with 3 s duration and 18–44 s intervals. The door was raised at the beginning of the shock, each trial was terminated when the mouse crossed into the nonshock compartment. Latency to escape and the number of escape failures were recorded automatically by software.

### 2.4. Stereotaxic Surgery and Microinjection

Mice were anesthetized and implanted with a guide cannula (3.3 mm) into lateral ventricle using a procedure described previously with some modifications [29]. The skull surface was first coated with 3% hydrogen peroxide. After the guide cannula was inserted into the lateral ventricle (coordinates: 0.6 mm posterior, 1.1 mm lateral, and −2.5 mm ventral to the bregma), Kerr Prime was applied onto the skull and cannula surface. Finally, the dental cement was used to fill the area around the cannula, and a dummy cannula was inserted into the guide cannula to maintain the cannula patency. Animals were individually housed, handled daily, and allowed to recover for 7 days after surgery.

All microinjections were performed on conscious, unrestrained, and freely moving mice in the cage. On the experimental day, a PE tubing connected to a 5 *μ*l syringe was inserted into the guide cannula and extended 1 mm beyond the tip. Drugs or vehicle was infused laterally in a volume of 3 *μ*l over 2 minutes. An additional 5 minutes was allowed for diffusion and prevention of backflow through the needle track before the injector was withdrawn. Mice were divided into four different treatment groups, including vehicle + saline, YJ + saline, YJ + NMDA, and vehicle + NMDA. Mice first received intracerebroventricular injection of NMDA (1 pmol/3 *μ*l) or vehicle and 30 minutes later were given intragastric administration of YJ (2 g/kg) or saline. Thirty minutes after YJ administration, mice were subjected to the OFT, TST, FST, and NSF.

### 2.5. Western Blot

The whole hippocampus was lysed in RIPA buffer containing protease inhibitors and phosphatase inhibitors. Protein concentration was determined colorimetrically by BCA assay (Pierce, Rockford, IL, USA). Protein lysates were separated by 12% SDS-PAGE electrophoresis and were transferred onto polyvinylidenedifluoride (PVDF) membranes. After blocking with 1% BSA for 1 hour, the membranes were incubated with primary antibodies. BDNF (SantaCruz Biotechnology, sc-546, 1 : 200), P-CREB (Cell Signaling Technology, 9198s, 1 : 500), CREB (Cell Signaling Technology, 9197, 1 : 500), PKA (Proteintech, 55388-1-AP, 1 : 1000), NMDAR1 (Cell Signaling Technology, 5104s, 1 : 1000), NR2A (Cell Signaling Technology, #4205, 1 : 1000), NR2B (Cell Signaling Technology, 4212s, 1 : 1000), and tubulin (Proteintech, 10094-1-AP, 1 : 2000) were used at 4°C overnight. The next day, blots were washed 3 times in TBST, followed by incubation with horseradish peroxidase-conjugated secondary antibodies for 1 hour. After the last wash for 3 times, the blots were visualized using the SuperSignal West Pico Chemiluminescent Substrate (Thermo Fisher Scientific Inc.). BDNF and pro-BDNF were normalized to tubulin bands, and P-CERB and total CREB bands were taken as a ratio of tubulin bands. All experiments were performed 3 times.

### 2.6. Statistics Analyses

Two-sample comparisons were carried out using two-tailed Student's *t*-test; multiple comparisons were made using one-way ANOVA, followed by the Bonferroni multiple comparison tests. Two-way ANOVA was used for the analysis of behavioral effects of NMDA, and Yueju in the TST, FST, and OFT. Analyses of variance with repeated measures were used in LH treatment at different time points. All data are presented as mean ± SEM, and statistical significance was accepted at the 5% level unless otherwise indicated.

## 3. Results

### 3.1. 2 g/kg Yueju Was Effective in Inducing Antidepressant Effect

**Figure 1:**
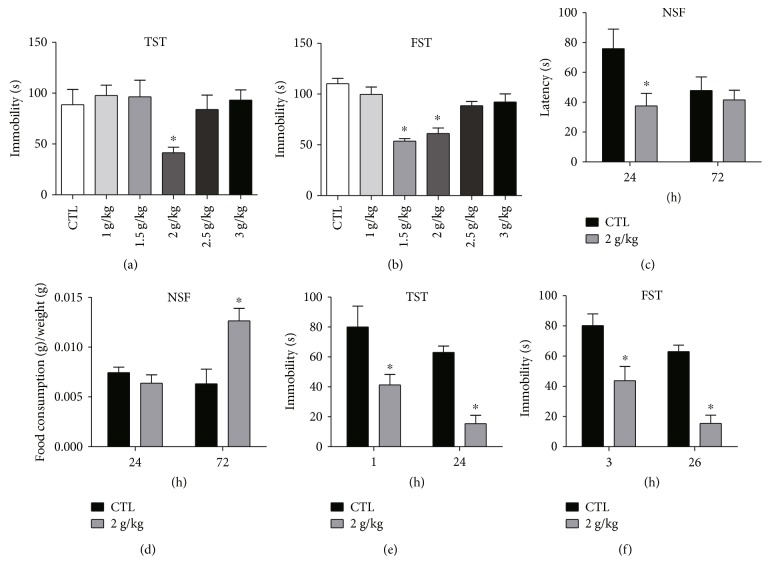
Screen of effective antidepressant dose of Yueju. The doses of YJ with 1 g/kg, 1.5 g/kg, 2 g/kg, 2.5 g/kg, and 3 g/kg were used for test. (a) There was significant treatment effects on tail suspension test (TST) performed 1 hour after a single administration of YJ (ANOVA, *F*(5, 51) = 2.918, *p* < 0.05) and (b) on forced swimming test (FST) carried out at 3 hours post administration (ANOVA, *F*(5, 49) = 15.05, *p* < 0.01). Mice were also tested for NSF at 24 h and 72 h after administration of 2 g/kg YJ (c, d). In the separate group of animals, animals were treated with 2 g/kg and tested with TST at 1 h and 24 h (e), as well as FST at 3 h and 26 h after administration YJ (f). Immobility time was measured for the last 4 min during the 6 min testing time for TST or FST. Data are means ± SEM. ^∗^*p* < 0.05, compared with control group.

The dose range from 1 g to 3 g/kg in mice was approximate to the routine use of Yueju nonprescriptively, and they were selected for testing antidepressant effect using TST, FST, and NSF. Only 2 g/kg of Yueju significantly reduced immobility time in the TST (*p* < 0.05) at 1 hour and FST (*p* < 0.05) at 3 hours post administration (Figures 1(a) and 1(b)). This dose of Yueju also reduced the latency to eat in NSF (*p* < 0.05, Figure 1(c)) at 24 hours and increased the food consumption (*p* < 0.05, Figure 1(d)) at 72 hours. Interestingly, it did not alter food consumption at 24 hours or latency at 72 hours (both *p* > 0.05). The dose of 1.5 g/kg effectively decreased the immobility time in the FST (*p* < 0.05) but not TST (Figure 1(b)). Collectively, 2 g/kg Yueju was an optimal dose that effectively elicits antidepressant response. To confirm the effect, an independent cohort of animals were tested for TST and FST quickly and one day after Yueju administration. The effect on TST (*p* < 0.05) at 1 hour and FST (*p* < 0.05) at 3 hours was replicated. Furthermore, the antidepressant effect was also detected at 24 hours for TST (*p* < 0.05) and 26 hours for FST (*p* < 0.05). Administration of Yueju did not affect the time spent in central area or total distance in the open field test (data not shown). Therefore, the dose of 2 g/kg was an effective dose and used in the following experiments.

### 3.2. An Intermittent Training following a 3-Day Training Period Resulted in a Long-Term Learned Helpless Activity

**Figure 2:**
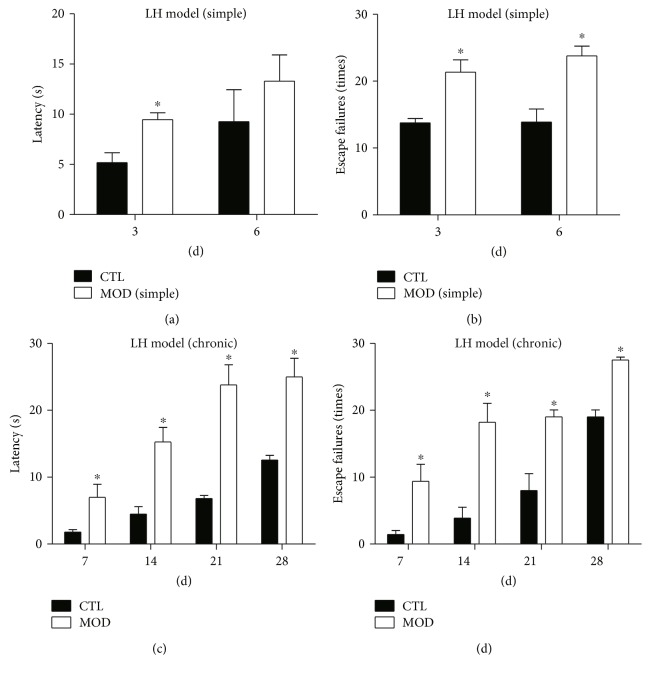
Duration of learned helpless response in two different models. On the top panel, escape failures and latency to escape was measured at 3 and 6 days post regular 3-day training paradigm (a, b). On the bottom panel, escape failures and latency to escape was measured weekly for 4 weeks following the 3-day plus 2 intermittent training paradigm (c, d). Control animals (CTL) received no training, MOD-simple group received 3-day training, and MOD received 3 days plus 2 intermittent training. Data are means ± SEM. ^∗^*p* < 0.05, compared with control group at the same time points, repeated measures, followed by *t*-test.

Firstly, we tested the persistent time of learned-helpless activity following the regular 3-day training paradigm. Animals were tested at 3 days and 6 days after the termination of training (Figures 2(a) and 2(b)). The repeated measures with the regular 3-day training did not show significant effects for time (*F*(1, 12) = 0.039, *p* > 0.1 for escape failure, *F*(1, 12) = 2.995, *p* > 0.1 for latency). The interaction between treatment and time had no difference (*F*(1, 12) = 0.852, *p* > 0.1 for escape failure, *F*(1, 12) = 0.013, *p* > 0.1 for latency). Post hoc analyses showed that both the latency to escape (*p* < 0.05) and frequency of escape failure (*p* < 0.05) was significantly different between the model and control animals at 3 days post training. However, after 6 days, only frequency of escape failure remained a deficit whereas there was no difference in latency to escape (Figures 2(a) and 2(b)), indicating that the learned helpless response has begun to diminish. To extend the duration of learned helplessness, a cohort of animals received two additional trainings 5 and 10 days posttermination of 3 days training. Animals were tested weekly until the fourth week post the last training (Figures 2(c) and 2(d)). The repeated measures showed significant effects for latency (*p* < 0.01) but not escape failure (*p* > 0.05). The analysis with the latency showed significant effects for time (*F*(3, 36) = 6.996; *p* < 0.05). Post hoc analyses showed that, from 7 to 28 days, both latency to escape and frequency of escape failure was significantly increased (*p* < 0.05), indicating the deficits lasted at least for 4 weeks.

### 3.3. Chronic Administration of Yueju Reversed the Learned Helplessness and Depressed Phenotype of Mice

**Figure 3:**
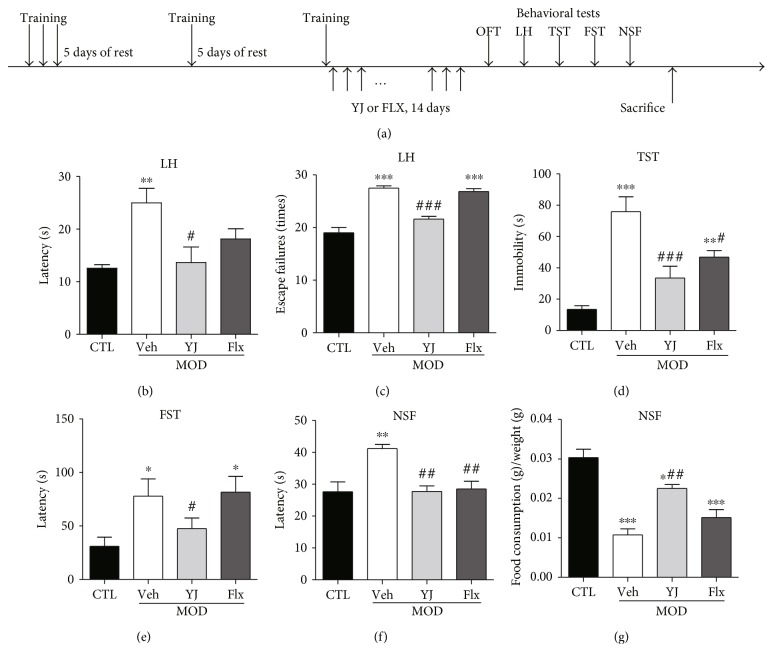
Behavioral effects after fluoxetine and Yueju treatment following chronic learned helplessness procedure. The timeline of the procedure is illustrated (a). Control animals (CTL) received vehicle treatment and no training, and animals exposed to chronic LH received administration of vehicle (Veh), Yueju (YJ), or fluoxetine (Flx) for 14 days. There were significant treatment effects on escape failures (ANOVA, *F*(3, 31) = 6.526, *p* < 0.05) and latency (ANOVA, *F*(3, 31) = 44.98, *p* < 0.05) (b, c). TST (ANOVA, *F*(3, 31) = 16.77, *p* < 0.05) and FST (ANOVA, *F*(3, 31) = 3.624, *p* < 0.05) were tested at 28 hours and 30 hours after drug treatment, respectively (d, e). NSF was tested at 48 hours after drug treatment. Food consumption (ANOVA, *F*(3, 31) = 23.23, *p* < 0.05) and latency (ANOVA, *F*(3, 31) = 8.73, *p* < 0.05) were measured for 10 minutes (f, g). ^∗^*p* < 0.05, ^∗∗^*p* < 0.01, ^∗∗∗^*p* < 0.001, compared to CTL; ^#^*p* < 0.05, ^##^*p* < 0.01, ^###^*p* < 0.001, compared to Veh.

Animals received 2 weeks administration of Yueju (YJ) or fluoxetine (FLX) following the chronic learned helpless paradigm and tested for LH, as well as OFT, TST, FST, and NSF (Figure 3(a)). The four groups have no difference in the total distance traveled or central time (data not shown). In the LH test, animals still demonstrated learned helpless behavior, and Yueju restored it in terms of both latency (*p* < 0.05 versus Veh) and escape failure (*p* < 0.001 versus Veh), without difference from the control group (Figures 3(b) and 3(c)). In contrast, chronic administration of fluoxetine failed to reverse either measurement in learned helplessness (both *p* > 0.05 versus Veh) and remained deficient (both *p* < 0.05 versus CTL). Additionally, animals also displayed other depressive responses, indicated by increase in immobility time in TST and FST, increased latency to eat in NSF, and reduced food consumption in NSF (all *p* < 0.05 versus CTL). Compared to the vehicle group, YJ significantly reduced the immobility time in TST (*p* < 0.001) and FST (*p* < 0.05), decreased latency to eat (*p* < 0.01), and increased food consumption (*p* < 0.01) in NSF. Chronic fluoxetine only restored TST (*p* < 0.05) and latency to eat (*p* < 0.01) in NSF. It did not improve the learned helplessness, neither did it alter immobility time in FST or food consumption in NSF (Figures 3(d), 3(e), 3(f), and 3(g)). In summary, repeated administration of 2 g/kg Yueju displayed full spectrum of antidepressant effect, whereas fluoxetine failed to improve the learned helplessness response, with success in only a minor part of the measurements.

### 3.4. Yueju Treatment Induced an Upregulation of BDNF Expression and PKA-CREB Signaling and Decreased the Upregulation of NR1, NR2A, and NR2B Expression in Learned Helplessness Mice

**Figure 4:**
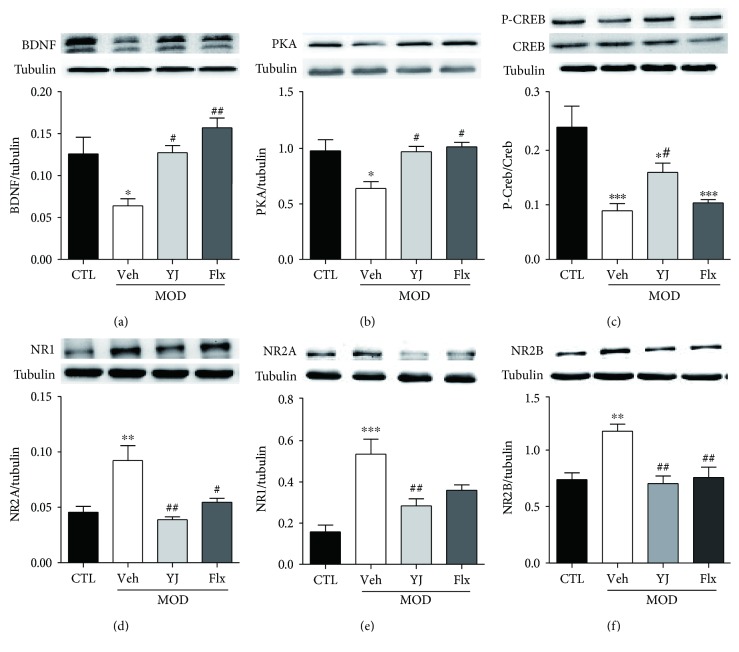
Western blots of CREB signaling and NMDA receptor subunits in the hippocampus in chronic LH animals receiving drug treatments. Mean ± SEM of protein expression levels of BDNF (ANOVA, *F*(3, 15) = 8.566, *p* < 0.05), PKA (ANOVA, *F*(3, 16) = 5.899, *p* < 0.05, P-CREB/CREB (ANOVA, *F*(3, 20) = 15.68, *p* < 0.05), NR1 (ANOVA, *F*(3, 16) = 10.54, *p* < 0.05), NR2A (ANOVA, *F*(3, 15) = 10.54, *p* < 0.05), and NR2B (ANOVA, *F*(3, 17) = 9.188, *p* < 0.05) in the hippocampus of CTL, Veh, YJ, and Flx groups. ^∗^*p* < 0.05, ^∗∗^*p* < 0.01, and ^∗∗∗^*p* < 0.001, compared to CTL; ^#^*p* < 0.05 and ^##^*p* < 0.01, compared to Veh.

The molecular signaling responsible for neural plasticity in the hippocampus associated with depression and antidepressant activity was examined. There was reduced signaling of PKA, CREB, and BDNF. Chronic Yueju restored CREB signaling (Figure 4(b), *p* < 0.05), whereas fluoxetine failed to alter it (*p* > 0.05). However, both Yueju and fluoxetine restored PKA and BDNF expression (Figures 4(a) and 4(c)). Additionally, there was significant increase in expression of NMDA subunits NR1, NR2A, and NR2B in the chronic learned helpless mice, which were reversed by either repeated treatment of Yueju or fluoxetine (Figures 4(d), 4(e), and 4(f)).

### 3.5. Inhibition of NMDA Signaling Was Required for the Antidepressant Effect of Yueju

**Figure 5:**
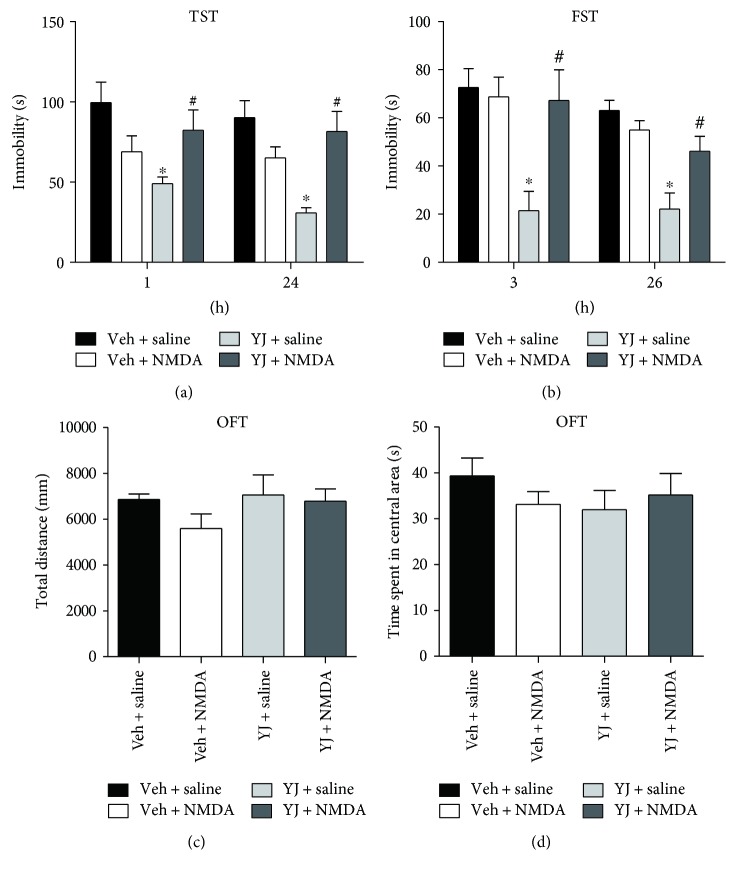
Intracerebroventricular microinjection of NMDA blocked Yueju (YJ) induced antidepressant effect. Mice received intracerebroventricular injection of vehicle or NMDA (1 pmol/site) 30 min prior to YJ (2 g/kg) or saline. TST was tested at 1 hour (two-way ANOVA, *F* = 4.476, *p* < 0.05) and 24 hours (two-way ANOVA, *F* = 6.572, *p* < 0.01) after the administration (a). FST was tested at 3 hours (two-way ANOVA, *F* = 4.783, *p* < 0.01) and 26 hours (two-way ANOVA, *F* = 12.472, *p* < 0.01) after the administration (b). OFT was performed 30 minutes post administration of YJ or Veh (c, d). ^∗^*p* < 0.01 compared with Veh-saline group; ^#^*p* < 0.05 compared with Veh-Yueju group.

The molecular analysis indicates the association of inhibition of NMDA receptor subunit expression with antidepressant effect of Yueju. To further assess the role of NMDA signaling in antidepressant effect of Yueju, animals were pretreated with NMDA at 30 minutes before YJ or vehicle. Only Yueju (*F*(1, 31) = 4.31, *p* < 0.05 for TST at 1 hour; *F*(1, 30) = 4.232, *p* < 0.05 for TST at 24 hours; *F*(1, 29) = 4.941, *p* < 0.05 for FST at 3 hours; *F*(1, 29) = 22.944, *p* < 0.05 for FST at 26 hours) but not NMDA (*F*(1, 31) = 0.079, *p* > 0.1 for TST at 1 hour; *F*(1, 30) = 1.333, *p* > 0.1 for TST at 24 hours; *F*(1, 29) = 2.846, *p* > 0.1 for FST at 3 hours; *F*(1, 29) = 2.522, *p* > 0.1 for FST at 26 hours) showed the main effect on TST or FST at each individual times. There was an approaching significant interaction between Yueju and NMDA treatment in the TST (*F*(1, 31) = 9.099, *p* < 0.01 at 1 hour; *F*(1, 30) = 14.411, *p* < 0.01 at 24 hours) and significant interaction in the FST (*F*(1, 29) = 8.001, *p* < 0.05 at 3 hours; *F*(1, 29) = 9.129, *p* < 0.01 at 26 hours). Post hoc analyses showed that microinfusion of NMDA did not alter the immobility time in TST (1 hour, *p* > 0.05; 24 hours, *p* > 0.05) or FST (3 hours, *p* > 0.05; 26 hours, *p* > 0.05) in animals receiving oral dose of vehicle. However, it blunted the reduction of immobility time in TST (1 hour, *p* < 0.05; 24 hours, *p* < 0.05) or FST (1 hour, *p* < 0.05; 24 hours, *p* < 0.05) by Yueju (Figures 5(a) and 5(b)). The four groups have no main effect in open field test for total distance (*p* > 0.05) and time spent in central area (*p* > 0.05) (Figures 5(c) and 5(d)).

## 4. Discussion

In this study, we first identified optimal dose of Yueju within the routine use range that conferred antidepressant activity. A chronic learned helplessness protocol was established to elicit a long-term depressive symptoms, which was used to test the therapeutic effect of repeated administration of Yueju or fluoxetine. Yueju reversed all behavioral deficits, in contrast to only partial recovery by fluoxetine. Interestingly, CREB activation was increased by Yueju but not fluoxetine, although they both normalized BDNF and PKA level. Yueju also attenuated the expression of NMDA subunits that was upregulated by the chronic LH. In agreement with it, NMDA pretreatment blocked the antidepressant effect of Yueju.

Previously, we found that relative high dose of ethanol extract of Yueju (equivalent to 10 g/kg raw herbal mixture followed by ethanol extract) alleviated the depressive symptoms in a rapid and lasting manner [30, 31]. The present study showed that 2 g/kg of the raw herbal mixture of YJ was also effective to elicit an antidepressant effect. However, the duration of the antidepressant effects lasted for less than 2 days after a single dose of 2 g/kg, whereas with high dose, the duration of antidepressant effect was as long as 5 days, much longer than the present dose in the same tests using same strain of animals [32]. Nonetheless, the routine dose of YJ is still significant for antidepressant effect. The long-term administration of the routine dose of Yueju is very safe, which is very important for the practice of clinical treatment of depression. Furthermore, this repeated Yueju was very efficacious to alleviate the long-term depressive symptoms that fluoxetine, a mainstream antidepressant, largely failed. The oral dose of fluoxetine for 14 days are sufficient to induce antidepressant effects when administered chronically in some animal models of depression [33]. However, some other studies showed no effects on some paradigm of depression in certain models or animal strains [34, 35]. This may reflect the fact to some degree that many patients are not responsive to conventional SSRIs including fluoxetine. Alternatively, this model may recapitulate the so called treatment-resistant depression. Nonetheless, routine dose of Yueju was found to be effective in the model that mimics the long-term depression and support the utility of Yueju on treatment of depression.

The present study developed and investigated the temporal profile of learned helpless response following a short training period. It has been shown that LH paradigm is an animal model widely used for the study of neural changes underlying behavioral phenotypes related to mood disorders [36, 37]. One day or two consecutive days after LH training, the depression phenotype duration was short in some strain of mice. Four consecutive days of induction training induced the phenotype of depression lasting for 1 week [38, 39]. Our data suggests that the depression phenotype already declined between 3 and 6 days after 3 consecutive training, but only two additional intermittent training evoked the depression behavior at least lasting for 28 days, which allows to evaluate the treatment effect on persistent depression status.

The present study examined several factors involving neural plasticity, but only found that CREB signaling was more closely related to the therapeutic effect of Yueju, as all other molecules examined including CREB activator PKA, downstream effector BDNF, and NMDA receptor subunits were similarly restored by fluoxetine as well. The decrease of CREB expression is associated with depression [40, 41]. Depressed rats with an overexpression of CREB in the dentate gyrus behaved similarly to rats treated with antidepressants [42]. Although the persistent antidepressant effect of a single high dose of Yueju was specifically dependent on PKA-CREB signaling [9], the restoration of CREB level in the present study was more likely contributed by chronic and accumulating effect of repeated low dose of Yueju. The crucial upstream and downstream signaling related to CREB for action of repeated routine dose of Yueju warrants further investigation.

Previously, it has been demonstrated that the antidepressant effect of a single high dose of Yueju is closely related to NMDA receptor subunit, especially NR1 [32, 43]. Dysfunction of NMDA receptors is implicated in mood disorders, demonstrating the importance of these receptors in depression [44, 45]. NMDA receptors consist of different subunits to form three subtypes: NR1, NR2, and NR3 [46, 47]. In the regular model of LH, the expression of NR1 subunit in the hippocampus also increased and sustained for several days, whereas a single high dose of Yueju reverses this effect in a persistent manner. In comparison, there was only temporal or no effect on NR2B or NR2A expression [32]. In this study, chronically learned helpless mice showed long-term abnormal upregulation of NR1, NR2B, and NR2A and repeated treatment of Yueju, as well as fluoxetine decreased their expressions, suggesting that the inhibition of NMDA signaling was associated with antidepressant action. The present study further demonstrated that inhibition of NMDA signaling is required by using intracerebroventricular pharmacological manipulation, confirming that NMDA signaling is an obligatory target of Yueju.

In summary, the present study demonstrated that the routine dose of Yueju has significant antidepressant efficacy and effectively attenuated behavioral deficits in chronic learned helpless animals. Restoration of neural plasticity via CREB signaling is crucially involved in the antidepressant effect of Yueju, whereas the inhibition of NMDA signaling is required and part of the mechanism of antidepressant efficacy of Yueju.
